# Soluble Klotho, a Potential Biomarker of Chronic Kidney Disease–Mineral Bone Disorders Involved in Healthy Ageing: Lights and Shadows

**DOI:** 10.3390/ijms25031843

**Published:** 2024-02-03

**Authors:** Julia Martín-Vírgala, Beatriz Martín-Carro, Sara Fernández-Villabrille, María Piedad Ruiz-Torres, Carlos Gómez-Alonso, Minerva Rodríguez-García, José Luis Fernández-Martín, Cristina Alonso-Montes, Sara Panizo, Jorge B. Cannata-Andía, Manuel Naves-Díaz, Natalia Carrillo-López

**Affiliations:** 1 Metabolismo Óseo, Vascular y Enfermedades Inflamatorias Crónicas, Instituto de Investigación Sanitaria del Principado de Asturias (ISPA), 33011 Oviedo, Spain; 2 Redes de Investigación Cooperativa Orientadas a Resultados en Salud (RICORS2040, Kidney Disease), 28040 Madrid, Spain; mpiedad.ruiz@uah.es; 3 Área 5—Fisiología y Fisiopatología Renal y Vascular del Instituto Ramón y Cajal de Investigación Sanitaria (IRYCIS), Physiology Unit, Department of Systems Biology, Facultad de Medicina y Ciencias de la Salud, Universidad de Alcalá, 28871 Alcalá de Henares, Spain; 4 Bone and Mineral Research Unit, Hospital Universitario Central de Asturias, 33011 Oviedo, Spain; 5 Nephrology Unit, Hospital Universitario Central de Asturias, 33011 Oviedo, Spain; 6 Department of Medicine, Universidad de Oviedo, 33011 Oviedo, Spain

**Keywords:** serum sKlotho, urinary sKlotho, CKD, CKD-MBD, biomarker, healthy ageing

## Abstract

Shortly after the discovery of Klotho, interest grew in its potential role in chronic kidney disease (CKD). There are three isoforms of the Klotho protein: αKlotho, βKlotho and γKlotho. This review will focus on αKlotho due to its relevance as a biomarker in CKD. αKlotho is synthesized mainly in the kidneys, but it can be released into the bloodstream and urine as soluble Klotho (sKlotho), which undertakes systemic actions, independently or in combination with FGF23. It is usually accepted that sKlotho levels are reduced early in CKD and that lower levels of sKlotho might be associated with the main chronic kidney disease–mineral bone disorders (CKD-MBDs): cardiovascular and bone disease. However, as results are inconsistent, the applicability of sKlotho as a CKD-MBD biomarker is still a matter of controversy. Much of the inconsistency can be explained due to low sample numbers, the low quality of clinical studies, the lack of standardized assays to assess sKlotho and a lack of consensus on sample processing, especially in urine. In recent decades, because of our longer life expectancies, the prevalence of accelerated-ageing diseases, such as CKD, has increased. Exercise, social interaction and caloric restriction are considered key factors for healthy ageing. While exercise and social interaction seem to be related to higher serum sKlotho levels, it is not clear whether serum sKlotho might be influenced by caloric restriction. This review focuses on the possible role of sKlotho as a biomarker in CKD-MBD, highlighting the difference between solid knowledge and areas requiring further research, including the role of sKlotho in healthy ageing.

## 1. The Klotho Protein Discovery: A Scientific Story

In 1997, during the generation of transgenic mouse lines, a Japanese research group led by M Kuro-o observed an unexpected complex ageing-like phenotype in one of the progenies, distinct from the anticipated transgenic mice. This coincidence led to the identification of a novel gene, named αKlotho, exhibiting ageing-suppressing actions. The intricate ageing-like phenotype in these mice included growth retardation, a shortened lifespan, osteoporosis, hypokinesis, atrophy of the genital organs, arteriosclerosis and ectopic calcification [1]. Subsequently, αKlotho was recognized as a co-receptor of the fibroblast growth factor 23 (FGF23) receptor [2], playing a role in phosphorus homeostasis [3]. Shortly after, the αKlotho gene was validated as an anti-ageing protein that, when overexpressed, extended the lifespan [4].

There are three isoforms of the Klotho protein: (1) αKlotho, (2) βKlotho and (3) γKlotho, encoded by three distinct genes [5]. (1) αKlotho is predominantly synthesized in the kidneys [5] and is present in the parathyroid gland [6,7], bones [8], choroid plexus [9], sinoatrial node [10], surface of immune cells [11] and the vasculature, although the latter is still a matter of controversy [12,13,14]. In the kidneys, αKlotho serves as a co-receptor of FGF23, a phosphaturic hormone, in the proximal tubules [5]. (2) βKlotho associates with the FGF21 receptor in the central nervous system and adipose tissue, inducing catabolic actions under situations of stress [15]. (3) γKlotho binds to the FGF19 receptor in the liver, inhibiting CYP7a1 hydroxylase expression and reducing bile acid synthesis [15,16].

This review will specifically focus on αKlotho, hereafter referred to as Klotho-, due to its potential relevance as a biomarker in chronic kidney disease (CKD).

## 2. Systemic Effects of Soluble Klotho

The transmembrane Klotho protein, which is mostly expressed in the kidneys, displays a three-domain structure: the intracellular, the transmembrane and the extracellular domains, the latter of which contains two sections, named KL-1 and KL-2 [17]. The extracellular domain can be secreted via transcytosis [18] or cleaved off by proteases such as a disintegrin and metalloprotease 10 (ADAM-10) and ADAM-17 [17], and released into the bloodstream and urine. This form of Klotho is called soluble Klotho (sKlotho), a 130 kDa protein that undertakes systemic actions independent of FGF23, many of them still poorly understood [18]. In addition, another form, a Klotho transcript produced by alternative splicing, is degraded before turning into an active protein [19]. Some studies have claimed that sKlotho carries out beneficial actions in different tissues, such as kidneys [20], vessels [21,22,23,24,25], endothelial cells and others [26,27]. Among the best-known beneficial actions of sKlotho are protection against vascular calcification due to the inhibition of phosphorus uptake [28] and its influence on the enzymatic activity that controls phosphorus transporters [29]. 

In addition, sKlotho can act at the cardiac level, inhibiting TRPC6 channels in cardiomyocytes, preventing stress-induced cardiac remodelling [21] and consequently having anti-hypertrophic effects [30]. It also reduced hyperphosphatemia-induced damage [31]. Endothelial cells also seem to be protected by sKlotho. In fact, in human umbilical vein endothelial cells (HUVECs), sKlotho inhibited tumor necrosis factor alpha (TNFα)-induced expression of two adhesion molecules, intercellular adhesion molecule 1 (ICAM-1) and vascular cell adhesion molecule 1 (VCAM-1), reducing endothelial inflammation [27]. Furthermore, it has been suggested that sKlotho is linked to vascular endothelial growth factor receptor-2 (VEGF-2) and transient receptor potential canonical 1 (TRPC-1) receptors in the endothelium, helping to maintain its integrity [32]. It can also reduce apoptosis and senescence acting through caspases 3 and 9 [26]. 

Additionally, sKlotho has demonstrated antioxidant activity in liver and brain tissue through apoptosis-signal-regulating kinase 1 (ASK-1) signalling [33,34]. In the bones of mice, sKlotho caused hypomineralization, probably through FGF23 signalling, regulating the expression of phosphate regulating endopeptidase homolog X-linked (PHEX), a protein that takes part in bone mineralization [35]. sKlotho also displayed anti-tumoral actions through insulin-like growth factor-1 (IGF-1) signalling, regulating ion channel expression [36,37] and acting as a ligand for monogangliosids at the plasma membrane [38]. In addition, it was found that sKlotho induced autophagy through the disruption of the Beclin-1- B-cell lymphoma 2 (Bcl2) complex, extending the lifespan in mice and protecting against age-related disorders, not only in the heart but also in the kidneys, where it was also found that sKlotho displayed anti-fibrotic actions by inhibiting transforming growth factor β1 (TGFβ1) [39] and Wnt signalling [25,40]. 

A key limitation on fully interpreting the systemic actions of sKlotho is that most studies have been carried out in different tissues, and so far, no receptor for sKlotho has been discovered. Thus, many mechanisms involved in the systemic actions of sKlotho are still unknown and further studies are needed in this area.

## 3. Soluble Klotho and CKD

Following the identification of sKlotho as a co-receptor for the FGF23 receptor in the kidneys, significant interest arose regarding its role in CKD [41], a condition resulting from various diseases, including diabetes, hypertension, glomerulonephritis, tubulointerstitial nephropathies and others [42]. CKD is often characterized as a silent disease, with symptoms generally mild or absent until advanced stages of the disease. However, several complications emerge in the early phases of CKD, including cardiovascular and bone disturbances, which have been grouped under the name of chronic kidney disease–mineral bone disorders (CKD-MBDs) [43]. This syndrome is frequently associated with reduced quality of life and a higher risk of mortality [44].

It is currently accepted that serum sKlotho levels decline during the progression of CKD [20,24,45,46,47,48], a trend similarly observed in urinary sKlotho, although the interpretation of the latter remains a matter of controversy [24,25,28,45,49,50,51]. Given that the kidneys constitute the main source of sKlotho, kidney damage itself is posited as a contributing factor to the low levels of sKlotho in CKD, providing the rationale of sKlotho as a potential biomarker of kidney deterioration. Various factors have been proposed to explain the decline of sKlotho in CKD, including high levels of albuminuria [50], hyperphosphatemia and epigenetic regulation of the Klotho gene promoter by inflammatory cytokines and uremic toxins [52]. Consequently, substantial uncertainty persists regarding the potential role of sKlotho as a reliable biomarker for CKD-MBD [53,54].

### Controversies and Limitations in the Measurement of Serum and Urinary Soluble Klotho

Numerous studies have presented conflicting or inconsistent findings regarding the suitability of serum and urinary sKlotho as biomarkers in CKD-MBD. One contributing factor to this inconsistency is the heterogeneity across studies on this topic. Many of these investigations have employed different experimental models of CKD or have included a limited number of CKD patients, in whom the kidney or vascular damage was poorly studied, leading to inconclusive results [45,46,47,53,55,56].

Another notable limitation is the lack of consensus on an appropriate standardized assay for assessing sKlotho. While ELISAs are commonly utilized, concerns have been raised about their precision and sensitivity [57]. Some reports advocate immunoprecipitation as the preferred reference technique for sKlotho assessment [58,59]. Despite immunoprecipitation being a precise technique, it is more complex, thus making it difficult to be incorporated as a routine technique in most clinical laboratories [59]. Additionally, there is no consensus on optimal procedures for extracting, processing and storing sKlotho samples to achieve optimal results [41,60]. This issue is particularly relevant when measuring urinary sKlotho, which is more unstable in urine than in serum [60]. Another limitation is the inability of available assays to distinguish between cleaved sKlotho, secreted sKlotho and KL-1 and KL-2 fragments [49]. Several of these technical problems may be overcome in the near future, as new assays and antibodies are emerging in the market, giving hope to this interesting area [61].

In summary, there is a great amount of research going on to improve the quantification of sKlotho, but still much information is lacking in regard to understanding and evaluating the potential usefulness of this protein as a reliable CKD-MBD biomarker.

## 4. Usefulness and Limitations of Measuring Soluble Klotho in Serum and Urine in the Diagnosis of CKD-MBD

CKD is frequently diagnosed in the middle or late stages of the disease when most of the important systemic damage associated with CKD-MBD is already established [44]. Consequently, the identification of serum or urinary biomarkers capable of detecting early damage is of great interest. 

Furthermore, the chronology of changes in various markers of CKD-MBD throughout the progression of CKD, including calcium, phosphorus, parathyroid hormone (PTH), calcidiol and calcitriol levels and FGF23, plays a crucial role in managing CKD-MBD. Since the discovery of sKlotho, several studies have tried to find (a) when sKlotho starts to change, (b) if the changes follow a particular pattern compared to other serum CKD-MBD biomarkers, (c) if serum and urinary sKlotho can be indistinctly used as biomarkers of sKlotho metabolism and (d) if the pattern of changes in serum and urine are similar. 

Several studies have demonstrated that serum sKlotho begins to decrease before serum FGF23 increases [25,58,62], and notably before the elevation of PTH [46,47,58,63]. Recent findings from our group in patients at stages CKD-2/3a to CKD-5 support this concept, highlighting serum sKlotho as the earliest serum CKD-MBD biomarker to change in CKD progression [25]. Unfortunately, it does not seem useful to follow the rate of progression of CKD-MBD, because it exhibits a mild and not clinically relevant further decrease as kidney function worsens. In fact, it did not show important changes throughout the progression of stages CKD-3b to CKD-5, a finding which is similar to what has been reported in previous clinical and experimental studies [47,62,63]. 

While these results do not position sKlotho as a precise biomarker for the degree of kidney damage, they support the concept that sKlotho is a useful biomarker for the beginning of CKD-MBD changes [25]. Nonetheless, there is no definitive agreement on the time course of serum sKlotho variations in the progression of CKD [46,53,55,64,65] and it is necessary to find explanations for the reported discrepancies. At least two factors could partially explain the lack of agreement observed among some studies: the misclassification of CKD stages and the different types of ELISAs used to measure sKlotho. 

Compared to serum sKlotho, there is limited research on urinary sKlotho in CKD, and most studies agree that urinary sKlotho declines early in CKD. Some studies suggest that urinary sKlotho correlates better with the decrease in glomerular filtration rate (GFR) than serum sKlotho, exhibiting a progressive decrease along the disease course [24,45,50]. On the contrary, recent studies have shown the opposite results [25], indicating that this topic is still a matter of debate and needs further investigation. One of the main difficulties in finding reasonable explanations for this controversy is the fact that the mechanism of removal of sKlotho through the urine is not yet fully clear. Two possible mechanisms have been proposed: tubular transcytosis and protease shedding [18,41,66]. Consequently, it remains challenging to position urinary sKlotho as an earlier and potentially more precise CKD-MBD biomarker than serum sKlotho. 

One important additional difficulty in evaluating the advantages and limitations of serum and urinary sKlotho is the limited number of studies assessing the relationship between their levels, making it challenging to obtain conclusive results [45,56,58]. Nevertheless, a recent study [25] addressed this topic, finding a significant relationship between serum sKlotho levels and GFR, which was not observed with urinary sKlotho. Further research is needed to fully understand and better interpret the value of serum and urinary sKlotho changes throughout the progression of CKD.

## 5. Soluble Klotho as a Biomarker of Cardiovascular and Bone Alterations

Low levels of serum sKlotho in CKD have been associated with cardiovascular disease and vascular calcification [67,68]. Furthermore, the decline of serum sKlotho in CKD has also been associated with carotid intima-media thickening [69], atherosclerosis [70,71], arterial stiffness [72] and hypertension [73]. However, this remains a controversial topic, as certain studies have not identified such associations in CKD [64,74,75,76,77]. Unfortunately, studies with urinary sKlotho are scarce, so the possible value of urinary sKlotho as a marker or predictor of cardiovascular abnormalities among the CKD-MBD setting has limitations.

The role of Klotho in bones has primarily been investigated through its mechanisms of action in mouse models. These studies revealed that Klotho, expressed in the osteocytes, might regulate bone formation and growth [78,79,80]. In fact, knock-out mice for the Klotho gene exhibit bone alterations which resemble human osteoporosis [1]. 

Recent clinical studies in CKD patients have found that lower levels of serum sKlotho are associated with bone impairment, particularly with altered trabecular microarchitecture [81]. Interestingly, the CARTaGENE health study showed that in CKD, both the lowest and the highest serum sKlotho levels were associated with fractures [82]. In other CKD settings, such as diabetic CKD [83] and renal transplant patients [84], lower serum sKlotho was also correlated with a higher risk of fractures and lower bone volume. 

Conversely, some studies have reported no association between serum sKlotho in CKD and bone structure, as measured by bone turnover [85] and bone mineral density [86]. The potential link between urinary sKlotho and bone alterations in CKD is even less clear than its connection with cardiovascular disorders. There are scarce experimental data, although it appears that in rats, low levels of urinary sKlotho could be associated with reduced trabecular bone density [87].

## 6. Healthy Ageing and Soluble Klotho

In recent decades, due to our increased life expectancy, the prevalence of accelerated-ageing diseases, such as CKD [88], neurodegenerative disorders [89] or cancer [90], has risen. These disorders involve several molecular mechanisms that accelerate tissular damage, leading to a diminished quality of life and premature death. Ageing inevitably leads to the heterogeneous but progressive loss of physical and mental capacities. To counteract these negative outcomes, adopting active lifestyles [91,92], engaging in social interactions [93,94,95] and adhering to caloric restriction diets [96] have been suggested to improve ageing-related disorders. Currently, exercise, social interaction and caloric restriction are considered key factors for healthy ageing and all have also been associated with sKlotho levels [97].

### 6.1. Exercise and Soluble Klotho

Several clinical studies have shown the beneficial effects of exercise in delaying ageing-related disorders, including fall risk [98,99,100], loss of muscle mass and strength, osteoporosis [101], cardiovascular disease [102], cognitive impairment and dementia [103], although the latter remains controversial [104].

Is sKlotho involved in these benefits? Unfortunately, studies focusing on the potential influence of serum sKlotho on the beneficial effects of exercise and vice versa, along with health outcomes, are scarce. Human pilot studies have examined serum sKlotho under different exercise conditions, revealing that exercise acutely increases sKlotho levels [105,106,107]. However, shortly afterwards, pre-exercise sKlotho levels were rapidly recovered [107,108]. Despite these limitations, the increase in sKlotho after exercise might exert positive effects in CKD patients, as high sKlotho levels have been associated with the attenuation of CKD progression [109] and higher bone mineral density [110].

While exercise appears to increase serum sKlotho, future studies in this area must consider the type [111,112] and duration [106,113] of the exercise, as well as the optimal time to assess sKlotho after exercising [107,108].

### 6.2. Social Interaction and Soluble Klotho

Ageing leads to the progressive loss of physical and cognitive function, including memory loss, hearing and sight impairment, reduced physical mobility [114] and a higher risk of developing neurodegenerative diseases [89], making social interaction harder to maintain and causing isolation and a lack of activity [114]. In fact, several studies have suggested that avoiding isolation and maintaining a socially active lifestyle might help to maintain self-autonomy, to reduce cognitive loss and preserve physical and mental wellness in the elderly [94,95,115,116]. 

Could age-driven dysfunctions be related to serum sKlotho? As previously discussed, the protein sKlotho is not only produced by the kidney but also by the choroid plexus in the central nervous system [1,9]. In fact, in Alzheimer’s disease, sKlotho has been found to be reduced in the cerebrospinal fluid [117]. In addition, in vivo experimental models have suggested that tissue-specific Klotho promotes brain cell maturation and myelinization [118] and regulates the inflammatory response in the central nervous system [9]. 

Recent studies have shown that low serum sKlotho was associated with worse cognitive performance, physical and psychological frailty, higher dependence on other persons in daily activities and increased frequency of falls [119,120,121]. 

In summary, low serum sKlotho seems to be related to poor cognitive and social interaction, but unfortunately, all data come from association studies and future research should focus more on causality.

### 6.3. Caloric Restriction and Soluble Klotho

Nearly a century ago, it was discovered that caloric restriction enhances longevity in rats [122]. Since then, numerous experimental studies have confirmed this discovery and sought to investigate whether this finding extends to humans, particularly in the context of accelerated-ageing diseases. In mice and monkeys, caloric restriction has been shown to promote longevity, improve health and reduce ageing-related disorders [123,124,125,126,127]. Caloric restriction also seems to extend the lifespan in humans, although it is challenging to perform long-term caloric restriction studies in humans due to difficulties in assessing whether participants adhered correctly to the diet [128]. In addition, genetic heterogeneity clearly influences the lifespan, thus probably altering the interpretation of the results. Supporting the positive effect of caloric restriction in humans, the CALORIE-2 clinical trial, a two-year caloric restriction study in healthy adults, found a reduction in body oxidative stress [129] and cardiometabolic risks [130], suggesting that the benefits of caloric restriction also apply to humans. 

Unfortunately, research on the potential effects of caloric restriction on Klotho is limited. Some animal models have shown that caloric restriction might increase Klotho protein expression in the kidneys [131] and hippocampus [132]. However, comparing these data is challenging due to differences in the tissues where Klotho was assessed and variations in the types of caloric restriction diets. 

A couple of clinical studies have explored the potential association of serum sKlotho with certain diet-related parameters. In women, a high energy, protein and carbohydrate intake, as well as a high visceral adiposity index (which assesses body fat distribution and dysfunction), were associated with low serum sKlotho levels [133,134]. However, there is currently no direct clinical data available on the possible effect of serum sKlotho levels and caloric restriction in the general population or in other diseases. These aspects should be addressed in future research.

## 7. Klotho as a Strategy for Treatment: Repletion of Soluble Klotho and Klotho-Inducing Drugs

Experimental studies in CKD have suggested that sKlotho could be replenished to reduce or prevent accelerated ageing. Several preclinical models have demonstrated some benefits of overexpressing or exogenously repleting sKlotho [24,135]. Transgenic mice in which Klotho was overexpressed displayed better kidney function, reduced vascular calcification [21,24,136] and less kidney damage [137]. Direct supplementation with recombinant sKlotho has been shown to protect against cardiac hypertrophy and fibrosis [138,139], reduce vascular and kidney calcification, increase mice lifespan [135] and prevent the impairment of blood pressure [140].

The epigenetic regulation of sKlotho could be another approach to restore its levels, allowing its endogenous reactivation [49,141,142,143]. Additionally, several experimental and clinical studies have showed that many CKD-related drugs might increase serum sKlotho or tissue Klotho, although the biological link between these drugs and Klotho remains to be elucidated. Some examples of Klotho-inducing drugs in CKD-related disease are the novel sodium glucose co-transporter type 2 (SGLT2) inhibitors [144], mammalian target of rapamycin (mTOR) inhibitors [145], statins [146], peroxisome proliferator activated receptor gamma (PPAR-γ) [147], vitamin D agonists [13,148] or angiotensin II receptor antagonists [149,150]. Gamma-aminobutyric acid (GABA) agonists have also been suggested to treat some CKD complications [151], but their influence on Klotho has been studied mainly in pancreatic tissue [152,153].

Unfortunately, there are no clinical trials that have tested the possible role and potential application of sKlotho as a therapy in humans [154]. Furthermore, the still-controversial results on the value of sKlotho as a biomarker of vascular and bone disorders [53,64] make the near future of sKlotho replacement strategies a wishful and desirable challenge, but still far away from the clinical grounds.

Thus, despite preclinical models offering promise, it is necessary to carry out clinical studies to determine if the replenishment of sKlotho could offer a feasible and effective approach to counteract sKlotho deficiency in ageing and CKD.

## 8. Concluding Remarks

**Figure 1:**
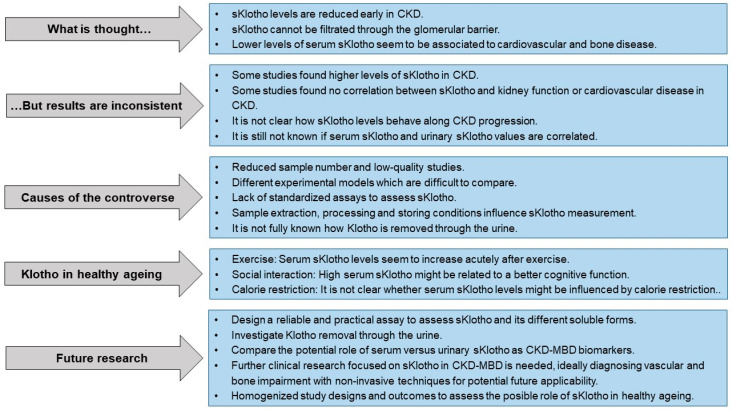
sKlotho “lights and shadows”.

The following sKlotho “lights and shadows” Figure 1 summarizes the facts and fancies, as well as our current knowledge on the role of this protein as a CKD-MBD biomarker and its possible association with healthy ageing.

Twenty-five years have passed since the anti-ageing gene Klotho was discovered. In that time, much research has focused on investigating the mechanisms of action of Klotho and its likely role as a biomarker in accelerated-ageing diseases. CKD is considered a reasonably effective model to study the vascular ageing process, and the kidneys are the main source of sKlotho; together, these two facts have put the spotlight on the potential role of sKlotho as a biomarker of CKD-MBD. However, the translational potential of sKlotho -as biomarker of CKD-MBD and its pharmacological use- is still incipient and further research is needed for a possible future applicability as a therapy.

## Data Availability

Not applicable.
